# Impact of preformed donor-specific anti-human leukocyte antigen antibodies on the clinical outcomes in pediatric liver transplantation patients: a retrospective cohort study

**DOI:** 10.3389/fimmu.2025.1703788

**Published:** 2026-01-09

**Authors:** Zhong-Yu Kang, Chun Liu, Wei Liu, Xueya Han, Daihong Li

**Affiliations:** Department of Blood Transfusion, Tianjin First Central Hospital, School of Medicine, Nankai University, Tianjin, China

**Keywords:** pediatric liver transplantation, DSA, graft loss, survival, antibody-mediated rejection, T cell-mediated rejection

## Abstract

**Purpose:**

The impact of donor-specific anti-human leukocyte antigen (HLA) antibodies (DSAs) in pediatric liver transplantation (LT) have been widely studied. The effect of preformed DSAs in pediatric LT is still controversial. This study aimed to evaluate the prevalence of preformed DSAs and their impact on clinical outcomes in pediatric LT recipients.

**Methods:**

This single-center retrospective cohort study included 243 pediatric patients who underwent LT between January 2019 and December 2022. The patients were divided into two groups according to the result of the pretransplant HLA antibody determined by Luminex™ Single Antigen Bead assay. The clinical outcomes, rejection, graft loss, patient survival, and the evolution of preformed DSAs were analyzed.

**Results:**

Thirty-one (12.7%) of the 243 patients had preformed DSAs, with most identified as class II DSAs. Among the patients with preformed DSAs, four had persistent preformed DSA, two had *de novo* DSAs, and 25 patients had cleared DSAs. Patients with and without preformed DSAs had comparable demographic characteristics but showed statistically significant differences by donor type (*p* = 0.042), intraoperative blood loss (*p* = 0.044), red blood cell transfusion (*p* = 0.006), and blood type (AB) (*p* = 0.035). Preformed DSAs were significantly associated with a longer intensive care unit stay (*p* = 0.008). No patients in either group developed antibody-mediated rejection post-transplantation. While the incidence of T cell-mediated rejection was higher in the preformed DSA-positive group compared to the negative group, this difference was not statistically significant (29.0% vs. 15.6%, *p* = 0.064). Other clinical outcomes and laboratory values were similar between the groups, with no association between preformed DSAs and overall graft loss or patient survival.

**Conclusion:**

This study showed that preformed DSAs were not associated with histologic or clinical outcomes in pediatric LT patients. Further studies with a larger sample size to obtain more reliable results are required to verify the risk associated with preformed DSAs in pediatric LT patients.

## Introduction

Pediatric liver transplantation (LT) has become a common therapeutic modality for children with end-stage liver diseases over the last decades, with reported excellent long-term patient and graft survival rates after pediatric LT (1–5). Preformed donor‐specific anti‐human leukocyte antigen (HLA) antibodies (DSAs) have been associated with lower graft survival and increased risk of acute or chronic rejection in patients who have undergone kidney, bowel, lung, pancreas and heart transplantation (6–10). Several studies have also demonstrated the roles of preformed DSAs in adult liver transplant recipients. In contrast to other organ transplants, the liver has been considered an immunologically privileged organ that is resistant to damage caused by DSAs; however, the impact of preformed DSAs in LT recipients requires further investigation (11–14). The liver widely expresses HLA class I and II antigens and can absorb class I and II DSAs. Additionally, the liver can secrete class I HLA antigens, which may also help in DSA clearance. Furthermore, Kupffer cells can eliminate class I HLA antigens that are soluble and coupled to DSAs by phagocytosing circulating DSAs (13).

The incidence of preformed DSAs in adult LT recipients detected by the single antigen bead assay is approximately 10–38% (15–17). In adult LT recipients, preformed DSAs are associated with a higher risk of chronic graft rejection and acute antibody-mediated rejection (AMR) (11, 16, 18). Other studies have also reported the association between preformed DSAs and impaired graft survival, graft fibrosis, and early patient mortality (17, 19, 20). Conversely, some studies reported that preformed DSAs were not correlated with graft function or graft survival, rejection, patient survival, or other clinical outcomes (21–23).

However, the role of preformed DSAs in pediatric LT recipients remains controversial. A retrospective single-center study found that the prevalence of preformed DSAs in pediatric LT recipients was 30%: preformed DSAs occurred in 39.3% of patients with T cell-mediated rejection (TCMR) and 25.7% of patients without TCMR, but the difference between the two groups was not statistically significant (24). Additionally, patients with preformed DSAs and *de novo* DSAs showed a significantly higher incidence of TCMR than patients without DSAs (24). But Grabhorn et al. found that pediatric patients with chronic rejection did not exhibit preformed antibodies (25).

This study aimed to determine the prevalence of preformed DSAs and to evaluate whether the preformed DSA was associated with biopsy-proven rejection, graft loss, and other clinical outcomes in pediatric LT recipients.

## Methods

### Patient population

**Figure 1:**
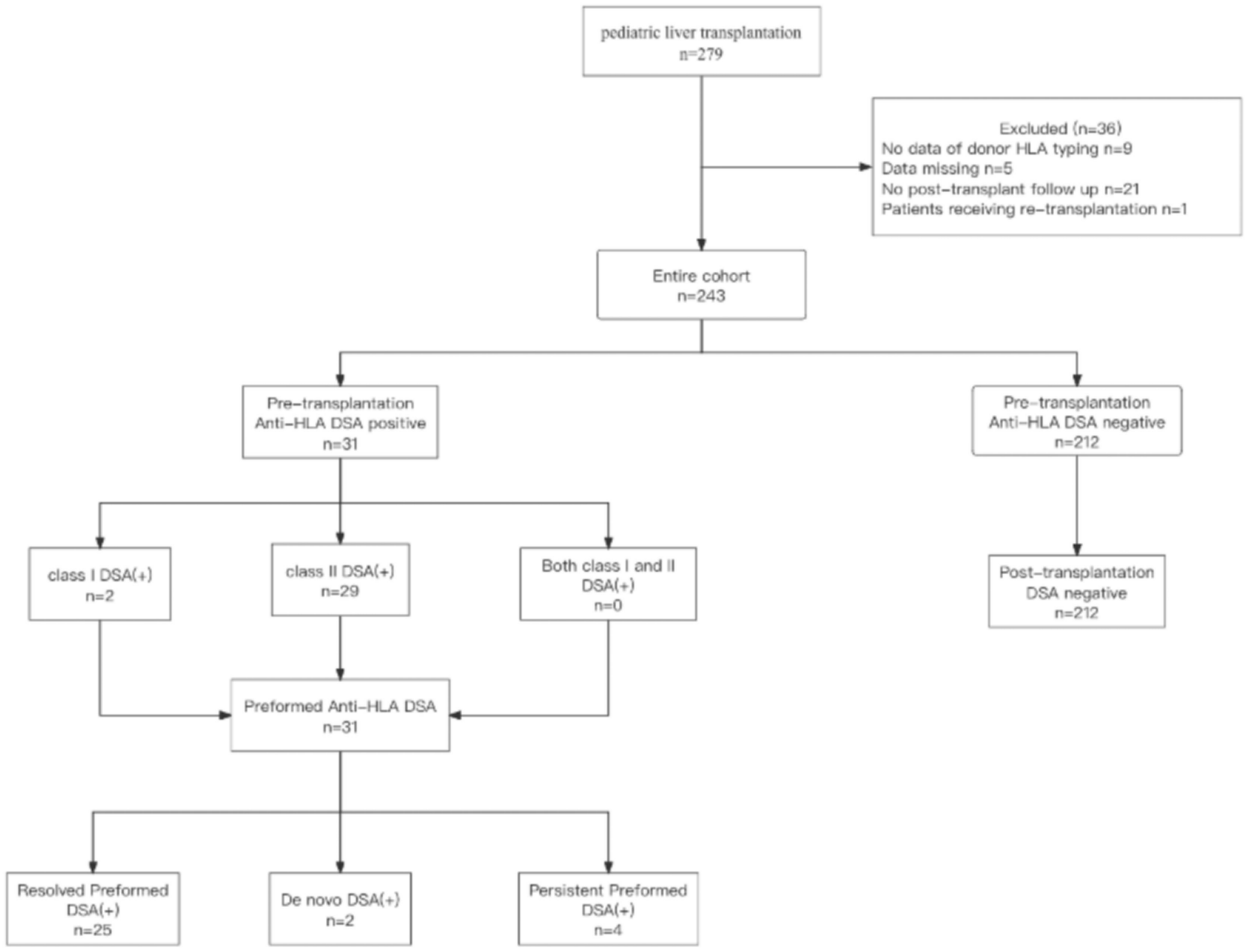
Flow chart detailing the selection of pediatric liver transplantation patients included in the study. HLA, human leukocyte antigens; DSA, donor-specific anti-human leukocyte antigens antibodies; DSA (+), DSA positive; n, number.

We conducted a single‐center retrospective cohort study involving 243 pediatric patients who had undergone a deceased or a living donor LT at Tianjin First Central Hospital from January 2019 to December 2022. We reviewed electronic medical records for clinical outcomes data. Demographic data, laboratory value, post-transplantation complications, graft survival, and patient survival were studied. Adult patients or patients without HLA DSAs assessed at transplantation were excluded from this study. The exclusion criteria included patients without donor HLA typing, and those who did not have post-transplantation data or had missing data (**Figure 1**). Patients who had previously undergone liver retransplantation were excluded, and only first liver transplants were included. All included patients were classified into two groups according to the presence of preformed DSAs before LT. We allocated patients into groups with and groups without preformed DSAs. Patients with preformed DSAs were then divided into resolved, persistent, and *de novo* DSA groups.

### HLA typing

HLA typing of all pediatric LT recipients and their corresponding donors included in this study was routinely performed by polymerase chain reaction (PCR) with a sequence-specific oligonucleotide probe using sequence-specific oligonucleotides (SSO) hybridization (LAB Type SSO) Typing Kits (One-Lambda, West Hills, CA, USA) on the Luminex platform to detect the specificity of HLA-A, -B, -C, -DRB1, -DPB1, and -DQB1. PCR-sequence-based typing (PCR-SBT) (Di Shuo Bacon Biotechnology) was used in the case of ambiguous or uncertain results.

### Detection of anti-HLA antibodies

Pre- and post-transplantation anti-HLA antibodies were detected in all patients using LIFECODES Life Screen Deluxe and LIFECODES LSA™ class I and II class based on Luminex^®^ technology (Immucor Transplant Diagnostics, Stamford, CT, USA), following the manufacturer’s protocol and analyzed with HLA FUSION software (One Lambda, Inc.). Antibodies detected with mean fluorescence intensity (MFI) above the threshold of 500 were defined as positive. MFI values <500 were regarded as negative, values of 500–4000 as weakly positive, values of 4000–10,000 as medium positive, and values >10,000 as strongly positive. Ethylenediaminetetraacetic acid was used during the experiments to eliminate the possible effects of complement interference or the prozone effect. Post-transplant DSA testing was routinely performed before transplantation and at approximately 3, 24, and 60 months after transplantation, as well as when graft dysfunction or rejection was clinically suspected. The 6-month interval mentioned in the Results reflects this early post-transplant monitoring window and corresponds to our center’s standard clinical practice.

### Immunosuppression protocol

The immune induction method consisted of intravenous methylprednisolone (10 mg/kg) during operation and basiliximab (10 mg) was given during surgery and postoperative day 4 (10mg for recipients weighing <35kg, 20mg for recipients weighing ≥35kg). For pediatric patients under the age of six and using monoclonal antibodies as immunological induction during the operation, tacrolimus was administered to patients 24 hours after transplantation to prevent rejection. For other patients (older than six years or who did not receive monoclonal antibodies as immunological induction intraoperatively), tacrolimus was used in combination with mycophenolate mofetil and methylprednisolone. Mycophenolate mofetil was given at a dose of 10 mg/kg/day in two separate doses and discontinuation at six months postoperatively. The dose of methylprednisolone was 5 mg/kg/day (maximum 100 mg/day) on the first postoperative day and was given in 2 to 4 injections, decreasing by 1 mg/kg/day (for patients <20 kg) or 20 mg (≥20 kg) per day. On the sixth operative day, the dose of methylprednisolone was changed to 4 mg (<40 kg) or 8 mg (≥40 kg) orally, with the dose halved at 1 month, halved again at 3 months, and discontinued at 6 months.

### Study outcomes and pathologic analysis

We compared the endpoints, including biliary complications, post-transplant infections, biopsy-proven rejection, liver fibrosis, graft loss of any cause, patient survival, and other components of the composite results between patients with or without preformed DSAs before transplantation. Biliary complications were defined as any biliary adverse event requiring radiological, endoscopic, or surgical intervention, including anastomotic or non-anastomotic biliary strictures and bile leaks, as identified by imaging or intraoperative findings (26, 27). Graft loss was defined as irreversible graft dysfunction resulting in re-transplantation or patient death, consistent with previous pediatric liver transplant literature (28, 29). Early mortality was defined as death from any cause within 90 days after transplantation, in accordance with established definitions from prior liver transplant outcome studies (30).

We investigated clinical outcomes throughout the observation period of this study. In our center, pediatric liver transplant recipients are typically followed several times during the first year after transplantation and then at regular intervals during long-term follow-up. The median follow-up duration was 28 months (range, 1–40 months), calculated from the date of transplantation to the last available clinical record documenting the patient’s status, such as DSA testing or other post-transplant follow-up assessments. The Desmet fibrosis score was used to evaluate graft fibrosis (31). Biopsies were performed when clinically indicated, and all reported rejection episodes were biopsy-proven. Rejection episodes, such as TCMR and AMR of the transplanted liver, were classified based on the Banff 2016 criteria for histopathologic classification (32).

### Statistical analysis

Statistical analysis was performed using SPSS 27.0 (IBM, Armonk, NY, USA), and GraphPad Prism 9 was used for data visualization. We used frequencies and percentages to describe categorical data. The differences between categorical variables in the preformed DSAs-positive group and the negative group were evaluated using Fisher’s exact and chi-squared tests. Continuous variables were summarized using the mean ± standard deviation (SD) and median with interquartile range (IQR). The Student t-test and Mann-Whitney U test were used to compare continuous variables between the two groups. P-values <0.05 were considered statistically significant.

## Results

### Patient characteristics

Of the 279 pediatric patients undergoing LT, 36 were excluded for the following reasons: 9 patients lacked donor HLA typing data, 21 patients were lost to follow-up post transplantation, 5 with data missing, and 1 patient underwent re-transplantation. The demographic and clinical characteristics of the remaining 243 included patients are summarized in **Table 1**.

**Table 1:** Demographic data of pediatric liver transplantation recipients by preformed DSA.

	All patients (n=243)	No DSA (n=212)	Preformed DSA (n=31)	*P*-value
Number of patients, n	243	212	31	
Recipient demographics				
Recipient’s gender, n (%)				
Male	111 (45.7%)	94 (44.3%)	17 (54.8%)	0.273
Female	132 (54.3%)	118 (55.7%)	14 (45.2%)	
Recipient’s age, median (IQR) (months)	8 (6-48)	8 (6-15)	10 (6-24)	0.304
Kasai operation, yes/no	102/141	87/125	15/16	0.439
Preformed antibodies, n (%)	31 (12.8%)	0 (0%)	31 (100%)	
Previous transplants, n (%)	0 (0%)	0 (0%)	0 (0%)	
Clinical diagnosis, n (%)				
Biliary atresia	210 (86.4%)	184 (86.8%)	26 (83.9%)	
Acute liver failure	1 (0.4%)	1 (0.4%)	0 (0%)	
Ornithine carbamoyl transferase deficiency	5 (2.1%)	5 (2.4%)	0 (0%)	
Crigler-najjar syndrome	2 (0.8%)	1 (0.4%)	1 (3.2%)	
Caroli’s Disease	1 (0.4%)	1 (0.4%)	0 (0%)	
Alagille syndrome	4 (1.6%)	3 (1.4%)	1 (3.2%)	
Cholestasis	3 (1.2%)	2 (0.9%)	1 (3.2%)	
Others	17 (6.9%)	15 (7.1%)	2 (6.5%)	
Donor demographics				
Donor gender, n (%)				
Male	118 (48.5%)	102 (48.1%)	16 (51.6%)	0.716
Female	125 (51.5%)	110 (51.9%)	15 (48.4%)	
Donor age, mean ± SD (years)	34 ± 8	31 ± 9	31 ± 9	0.804
Liver transplant donor type, n (%)				
Parent donor	209 (86%)	186 (87.7%)	23 (74.2%)	0.042
Deceased donor	34 (14%)	26 (12.3%)	8 (25.8%)	
Operation profiles				
Cold ischemic time, median (IQR)	74 (55-94)	74 (55-94)	78 (53-108)	0.672
Intraoperative blood loss (mL)	300 (200-400)	400 (250-500)	300 (200-400)	0.044
Intraoperative transfusion				
RBC transfusion (u)	2 ± 1	2 ± 1	3 ± 2	0.006
Fresh−frozen plasma, n (%)	112 (46.1%)	94 (44.3%)	18(58.1%)	0.152
Graft type, n (%)				
Reduced liver	235 (96.7)	205 (96.7%)	30 (96.8%)	0.982
Full size	8 (3.3%)	7 (3.3%)	1 (3.2%)	
Blood type, n (%)				
A	78 (32.1%)	67 (31.6%)	11 (35.5%)	0.087
B	60 (24.7%)	53 (25%)	7 (22.6%)	0.122
O	79 (32.5%)	66 (31.1%)	13 (41.9%)	0.393
AB	26 (10.7%)	26 (12.3%)	0 (0%)	0.035
ABO, n (%)				
Compatible	220 (90.5%)	192 (90.6%)	28 (90.3%)	0.966
Incompatible	23 (9.5%)	20 (9.4%)	3 (9.7%)	
Preoperative blood test				
TBIL (μmol/L)	188 (53-302)	255(32-370)	186 (57-300)	0.393
DBIL (μmol/L)	160 (43-249)	212 (16-270)	160 (48-246)	0.650
IBIL (μmol/L)	23 (7-46)	29 (9-55)	23 (7-43)	0.359
ALT, U/L	122 (76-202)	124 (80-202)	105(40-220)	0.387
AST, U/L	185 (111-311)	186 (112-302)	181 (80-390)	0.778
ALP, U/L	581 (354-872)	669 (360-1037)	575 (352-820)	0.281
GGT, U/L	249 (104-614)	249 (104-618)	260 (94-486)	0.781
Albumin (g/L)	35 ± 6	35 ± 6	34 ± 5	0.117
HGB (g/L)	97 ± 18	98 ± 19	93± 16	0.165

DSA, donor specific antibody; LT, liver transplantation; IQR, interquartile range; SD, Standard deviation; TBIL, Total bilirubin; DBIL, Direct bilirubin; IBIL, indirect bilirubin; ALT, alanine transaminase; AST, aspartate transaminase; ALP, alkaline phosphatase; GGT, Gamma-glutamyl transferase; Alb, albumin; Hgb, Hemoglobin.

Of these 243 included patients, 31 (12.8%) had preformed DSAs before LT and 212 (87.2%) patients were negative for preformed DSAs (**Figure 1**). Compared to patients without preformed DSAs, those with preformed DSAs had a higher incidence of parent donor type, greater intraoperative blood loss, and received more blood transfusion therapy during the operation, and were more frequently had an AB blood type. There were no significant differences between the other characteristics of patients with or without preformed DSAs.

### Donor‐specific antibodies

Of the patients with preformed DSAs, 25 (80.6%) had resolved preformed DSAs, 4 (12.9%) patients with persistent preformed DSAs and 2 (6.5%) patients developed *de novo* DSAs after transplantation (**Figure 1**). Of the 31 patients with preformed DSA, 2 (6.5%) patients had only class I DSAs (1 patient with antibodies against HLA-A and the other with antibodies against HLA-B) and 29 (93.5%) patients had only class II DSAs(17 patients with HLA-DR antibodies; 2 patients with HLA-DP antibodies; 10 patients with HLA-DQ antibodies), and no patients had both class I and class II DSAs. The MFI values were 1491 for preformed HLA-A DSA and 1107 for preformed HLA-B DSA. The median MFI for HLA-DR DSA was 5228 (range: 2676–13874), with a mean level of 1617± 236, and 2007 for HLA-DQ DSA (range: 1643–5360). **Table 2** shows the specificity and MFI of preformed DSAs for pediatric patients.

**Table 2:** Human leucocyte antigen-antibody status of included pediatric liver transplantation patients during cross-sectional study period.

Cases	n (%)
Pre-DSA positive	31
Class I	2 (6.5%)
DSA-A	1 (3.2%)
MFI DSA HLA-A class I	1491
DSA-B	1 (3.2%)
MFI DSA HLA-B class I	1107
Class II	29 (3.5%)
DSA-DRB1	17 (54.8%)
MFI DSA HLA-DRB1 class II, median (range)	5228 (2676-13874)
DSA-DPB1	2 (6.5%)
MFI DSA HLA-DRB1 class II, mean ± SD	1617 ± 236
DSA-DQB1	10 (32.3%)
MFI DSA HLA-DQB1 class II, median (range)	2007 (1643-5360)
Class I and II	0 (0%)

MFI, mean fluorescence intensity.

### Post-transplantation evolution of preformed DSA

**Figure 2:**
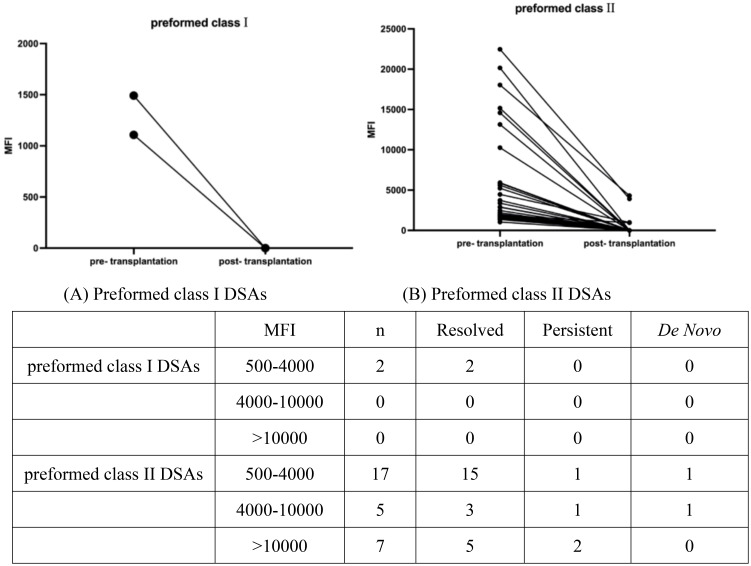
Characteristics of **(A)** preformed class I DSAs and **(B)** preformed class II DSAs in pediatric liver transplantation patients, including immunizing events and DSA specificities, as well as the evolution of the DSA MFI status before and after transplantation.

**Figure 3:**
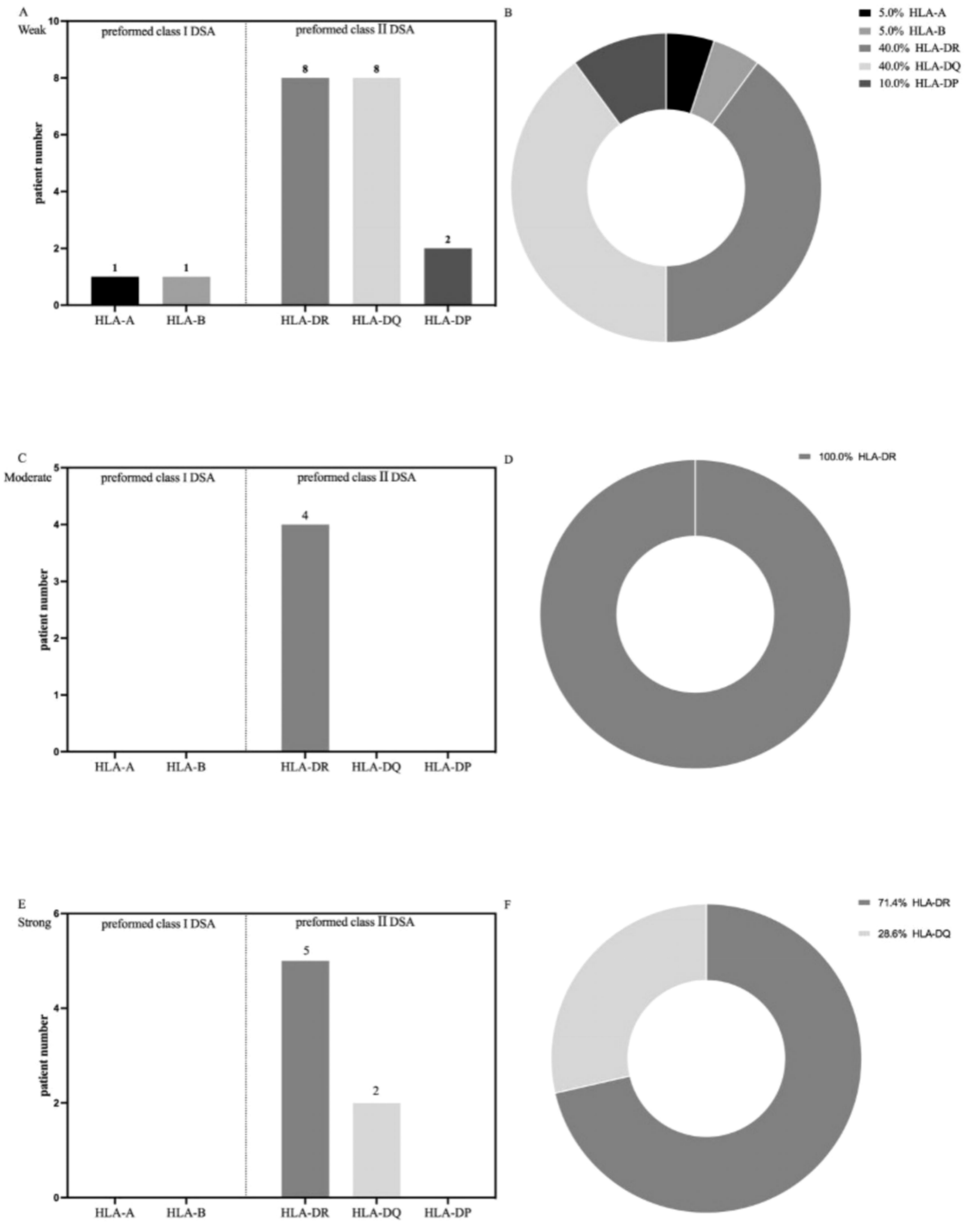
Immunological characteristics of preformed HLA-DSA by class and specificity. **(A–F)** Pie graphs represent the percentage of HLA-DSA specificity of the HLA-DSA group before liver transplantation.

**Figures 2** and **3** depict the characteristics of preformed DSAs and present the strength, class, specificities, and evolution of MFI values of preformed DSAs post-transplantation. The MFI strength of preformed DSAs was related to its class and specificity. Class I preformed DSAs were weakly positive, with an MFI of 1000–4999, and resolved in both patients after transplantation. Among the 29 patients with class II preformed DSAs, 17 had weak positive levels (MFI: 500–4000), 5 had medium positive levels (MFI: 4000–10000), and 7 had strong positive levels (MFI >10000). Of these, 23 patients (79.3%) showed complete disappearance of DSAs within six months after transplantation, including 15 with weak, 3 with moderate, and 5 with strong positive MFIs. Four patients (13.8%) had persistent DSAs during the same period, and two additional patients (6.9%) developed *de novo* DSAs (one with MFI 500–4000 and one with MFI 4000–10000). Among the two patients with *de novo* DSAs, one became antibody-negative within 12 months after transplantation, while the other was not re-tested after the 3-month time point. Neither of them showed clinical or biochemical evidence of graft dysfunction during follow-up.

Regarding antibody specificities, two patients had DSAs against HLA class I (one with anti-HLA-A antibodies and another with anti-HLA-B antibodies) and 18 patients had DSA against HLA class II (8 with anti-HLA-DR antibodies, 8 with anti-HLA-DQ antibodies, and 2 with anti-HLA-DP antibodies) with MFI values between 500 and 4000. All medium HLA-DSAs accounted for the anti-HLA-DR antibodies. Seven patients had DSAs with a cumulative MFI value >10000, 5 had anti-HLA-DR antibodies, and 2 had anti-HLA-DQ antibodies.

### Clinical outcomes

We evaluated the clinical outcomes after transplantation of recipients with and without preformed DSAs (**Table 3**). Our study showed that patients with preformed DSAs had significantly longer ICU stay than those without DSAs (p = 0.02). There were no significant differences in post-transplantation complications, including biliary and vascular complications, biliary infection, infectious complications (cytomegalovirus and Epstein–Barr virus infections), pulmonary infection, or alteration of intestinal flora. The incidence of bacterial and fungal infection was not significantly different between patients with and those without preformed DSAs during the follow-up period. Additionally, no patient presented with AMR post-transplantation. There was no statistical difference between the groups with respect to laboratory findings including liver function test results. Patients with and those without preformed DSAs had a similar incidence of graft loss and survival. Among the included patients, two experienced graft loss and one died within 90 days after transplantation (on postoperative day 23), corresponding to an early mortality rate of 1.4%. These three events occurred in different individuals. The death was not related to graft failure, and subsequent follow-up data for the two graft loss cases were unavailable.

**Table 3:** Clinical and pathological outcomes.

	All patients (n=243)	No DSA (n=212)	Preformed DSA (n=31)	*P*-value
Post-transplant events				
ICU stay (d)	2 (2-3)	2 (2-3)	3 (2-4)	0.008
Hospital stays (d)	26 (20-34)	22 (19-27)	28 (17-36)	0.116
Biliary complications, n (%)	4 (2.1%)	3 (1.4%)	2 (6.5%)	0.065
Biliary infection, n (%)	9 (3.7%)	9 (4.2%)	0 (0%)	0.242
Vascular complications, n (%)	34 (14.0%)	31 (14.6%)	3 (9.6%)	0.458
Cytomegalovirus infection, n (%)	37 (15.2%)	30 (14.1%)	7 (29.2%)	0.222
Epstein-Barr virus infection, n (%)	84 (34.6%)	71 (33.5%)	13 (41.9%)	0.356
Pulmonary infection, n (%)	13 (5.3%)	11(5.2%)	2 (6.5%)	0.581
Alteration of intestinal flora, n (%)	10 (4.1%)	9 (4.2%)	1 (3.2%)	0.790
Antibody mediated rejection, n (%)	0 (0%)	0 (0%)	0 (0%)	n.a.
T-cell-mediated rejection, n (%)	42 (17.1%)	33 (15.6%)	9 (29.0%)	0.064
Graft loss, n (%)	2 (0.8%)	1 (0.4%)	1 (3.2%)	0.113
Patient death, n (%)	1 (0.4%)	1 (0.4%)	0 (0%)	0.702
Bacterial infection, n (%)	15 (6.2%)	12 (5.7%)	3 (9.7%)	0.385
Fungal infection, n (%)	1 (0.4%)	1 (0.4%)	0 (0%)	0.702
Fibrosis, n (%)	51 (20.9%)	48 (22.6%)	3 (9.7%)	0.098

n.a., not available; Early mortality was defined as death within 90 days after transplantation.

### T cell-mediated rejection

Of the 243 patients, 42 were diagnosed with TCMR: 9 recipients with preformed DSAs and 33 recipients without preformed DSAs. There were no statistically significant differences in rates of TCMR between the two groups, although rate was higher patients with preformed DSAs (29%) compared to those without preformed DSAs (15.5%) (**Table 3**, p = 0.064).

### Discussion

In this retrospective study, we investigated the frequency of preformed DSAs in pediatric LT recipients and assessed whether the presence of preformed DSAs was a risk factor for adverse clinical outcomes. The findings showed that the frequency of positive preformed DSAs in our cohort was 31/243 (12.8%), and preformed DSAs were not significantly correlated with the development of adverse clinical outcomes post-transplantation.

In contrast to other organs, the liver was considered an immunologically privileged transplanted organ, as it is more resistant to damage caused by DSAs, primarily due to its ability to absorb antibodies (27–29). Several previous studies have shown a strong association between the presence of preformed DSAs and an increased risk of rejection and inferior graft survival outcomes in heart, lung, and kidney transplant patients (9, 33, 34). Moreover, in LT, preformed DSAs are associated with an increased risk of graft rejection, graft fibrosis, early mortality, and decreased graft survival (11, 23, 35, 36). However, the roles of preformed DSAs in pediatric LT remain controversial. Limited studies have assessed the prevalence of preformed DSAs in pediatric LT patients, and the relationship between preformed DSAs and graft rejection, patient survival, and other clinical outcomes remains underexplored.

Previous studies showed that the prevalence of preformed DSAs in adult patients undergoing living donor LT varies between 10% and 38% (17, 34). In the present study, the frequency of preformed DSAs in pediatric patients undergoing LT was 12.8%, consistent with the findings of other studies. However, Schluckebier et al. reported that 31.7% of pediatric LT patients had at least one preformed DSA with an MFI value exceeding 1000 for class I or class II DSAs (24). The reasons for this difference remain unclear; we hypothesize that it may be primarily due to differences in MFI cut-off values, which range from 500 to 2000 across different laboratories, as well as the inclusion of heterogenous populations with varying underlying causes of liver disease included in the studies (35, 36).

The findings of this study indicated a significant difference in the incidence of parent donor type and AB blood type between patients with and those without preformed DSAs. Additionally, patients with preformed DSAs had more intraoperative blood loss and received more blood transfusion therapy during the operation than those without preformed DSAs. Our data also suggested that, in contrast to recipients without preformed DSAs, preformed DSAs were not associated with clinical outcomes and the occurrence of AMR. Shizuku et al. found that 19.2% of patients had preformed DSAs, and there were no significant differences in the incidence of cytomegalovirus infection, bacteremia, patient survival, and pathology findings between groups with and without preformed DSAs (32). In accordance with our findings, Del Bello et al. demonstrated that liver transplant patients with preformed DSAs had similar incidences of biopsy-proven liver rejection and survival (37). Furthermore, Del Bello et al. did not find any impact of preformed DSAs on patient survival (23). Other studies have demonstrated no significant associations between preformed DSAs with an increased risk of bile duct lesions, graft dysfunction, acute rejection, and graft loss (21, 22, 36, 38).

In contrast, several studies have reported that preformed DSA is a risk factor for graft rejection, graft loss, and death. Goto et al. found that higher DSA‐MFI values for preformed DSAs were associated with worse graft outcomes in living donor LT (37). Legaz et al. demonstrated a significant difference in the incidence of graft survival among patients with preformed DSAs compared to the other group (36). Del Bello et al. found that high-MFI preformed DSAs were associated with graft rejection after LT (23). Tamura et al. performed a retrospective study and found that preformed DSAs were associated with elevated 90-day mortality and a higher incidence of ACR in LDLT recipients (17). Additionally, preformed DSAs have been associated with worse graft outcomes, such as higher rates of AR, graft failure, and death (38–43). Caillard et al. assessed the evolution of preformed DSAs in kidney transplant recipients and found that persistent preformed DSAs were associated with a higher risk of graft loss and development of AMR post-transplantation (38). These results are in contrast with the findings of the present study, which showed that all the MFI values declined post-transplantation, and only four patients had persistent preformed DSAs. The difference between our results and those of other studies could be explained by the liver’s ability to absorb antibodies.

This study also suggested that compared to recipients with preformed DSAs, those with preformed DSAs did not show a higher risk of TCMR. These results are consistent with the findings of previous studies, showing that preformed DSAs were not associated with a higher risk of TCMR. In pediatric LT, Schluckebier et al. reported that preformed DSAs had no negative association with the development of TCMR post-transplantation compared to the group without preformed DSAs (24). Taner et al. found that most preformed DSAs resolved after LT, and the incidence of acute cellular rejection between the two groups was not significantly different (16). Kim et al. also demonstrated that the occurrence of acute cellular rejection was similar between the two groups after transplantation (22). In contrast, Tamura et al. found significantly higher proportions of TCMR in patients with preformed DSAs compared to those without preformed DSAs (17). Musat et al. reported that pre-transplant DSAs assessed by SAB were significantly associated with ACR (43).

Our study has several limitations that must be acknowledged. First, this was a retrospective single-center cohort study, which could not exclude some potential bias. Hence, a large prospective large multicenter study is needed to obtain more reliable results. Second, we did not evaluate the impact of resolved, persistent, and *de novo* performed DSAs on clinical outcomes in pediatric LT patients due to the small sample size of this study. Third, although we observed a relationship between preformed DSAs and TCMR development in pediatric liver transplant recipients, we could not perform other subgroup analyses based on DSAs MFI values due to small sample sizes. Fourth, we cannot evaluate the role of preformed HLA-Cw DSAs in pediatric LT patients because the positive preformed DSAs included in our cohort were HLA- A, B, DR, DP, and DQ DSA. Fifth, our data did not highlight the specific detrimental effects of HLA- A, B, DR, DP, and DQ preformed DSAs due to the small number of patients with preformed DSAs. Sixth, we did not test the complement-binding or Ig subclasses of the preformed DSAs.

## Conclusion

This study demonstrated that preformed DSAs have no statistically significant impact on pediatric patient survival, AMR, TCMR, graft loss, and other clinical outcomes. However, given the small number of patients with preformed DSAs in this study, further large cohort studies with long-term follow-up are needed to determine whether preformed DSAs have an adverse effect on pediatric LT.

## Data Availability

The datasets presented in this study can be found in online repositories. The names of the repository/repositories and accession number(s) can be found in the article/supplementary material.
